# Implicit signatures of voluntary action reduce with repeated motor practice

**DOI:** 10.1007/s00221-023-06675-w

**Published:** 2023-08-24

**Authors:** Harriet Dempsey-Jones, Bartosz Majchrowicz, Patrick Haggard

**Affiliations:** 1 Institute of Cognitive Neuroscience, University College London, London, UK; 2 Institute of Psychology, Jagiellonian University, Krakow, Poland; 3 School of Psychology, University of Queensland, Brisbane, Australia

**Keywords:** Intentional binding, Voluntary action, Agency, Motor learning, Training, Automatization

## Abstract

The sense of controlling one’s actions and their consequences is a critical aspect of successful motor activity. While motor performance typically improves with learning, it is unclear whether, how, and why higher order aspects of motor cognition are also affected. Here, we used an implicit measure of sense of agency—the ‘intentional binding’ effect—as participants learned to make a skilled action involving precise control of thumb adduction. These actions were predictably followed by a tone (the outcome). At pre-test, we showed the perceived time of the tone was shifted towards the thumb action, compared to a control condition in which tones occurred without actions. Next, a *relevant training* group learned to refine the direction of the thumb movement, while an *irrelevant training* group was trained on another movement. Manipulation checks demonstrated that, as expected, the relevant training group improved performance of the trained movement, while the irrelevant training group did not. Critically, while both groups still showed binding of the tone towards the thumb action at post-test, the relevant training group showed less binding than the irrelevant training group. Given the link between intentional binding and volitional control of action, we suggest our result demonstrates subjective agency over the outcome of a skilled action decreases as practice makes the skilled action more fluent. We suggest that this reduction in sense of agency over movement outcomes is consistent with the decreasing cognitive engagement, or automatization, that occurs during skill learning.

**Supplementary Information:**

The online version contains supplementary material available at 10.1007/s00221-023-06675-w.

## Introduction

The process of learning a motor skill consists of three distinct phases. First, an attentionally demanding initial phase, then an intermediate phase with more stable performance levels, and a final automatic (overlearning) phase—where learning reaches an asymptote (reviewed in Luft and Buitrago 2005; Halsband and Lange 2006). While there is good knowledge regarding how motor kinematics change with practice (e.g. Debaere et al. 2004), and a developing understanding of the neurocognitive networks engaged (Puttemans et al 2005; see Sect. “Discussion”), there is relatively little information about how higher order aspects of motor control change as learning progresses.

One such higher order property is the sense of agency: the feeling of being in voluntary control of one’s own actions, and therefore of those actions’ external outcomes (Haggard 2005, 2008). Many theories link sense of agency to motor prediction. Using an internal model of the motor system, an agent can predict the likely consequences of their own motor commands. If the predicted consequences are subsequently reported by the senses, the agent knows that the corresponding events were caused by their own actions (Frith et al. 2000). Thus, a subjective sense of agency is generated by the computational models used to plan and execute skilled actions (Haruno et al. 2001). These models are acquired through feedback error learning (Kawato 1999). This suggests the sense of agency might vary during the process of motor skill learning. The current study investigated this possibility. We used an implicit paradigm widely proposed to measure the subjective experience of the link between volitional actions and their outcomes, known as *intentional binding*. This measure has been proposed as an implicit proxy for the sense of agency (Haggard et al. 2002).

In the intentional binding paradigm, a simple action (e.g. a keypress) reliably triggers an outcome (e.g. a tone). This pairing causes shifts in the perceived time of both the action and its outcome. Specifically, action and outcome are drawn towards each other in time (reviewed in Haggard 2005, 2008; Moore and Obhi 2012). That is, the keypress is perceived to have occurred later, and the tone earlier, compared to when either action or tone occurs alone. If the same finger movement is made passively (e.g. evoked by magnetic brain stimulation), this temporal attraction is absent or even reversed (Haggard et al. 2002).

Some research has questioned the link between intentional binding and the sense of agency, proposing that the actual driving mechanism of intentional binding is not one’s own specific agency but rather perceptions of causality in general (Buehner and Humphreys 2009). Indeed, temporal binding can also be present in passively observed rather than actively executed movements, provided experimental conditions control for all information except internal signals (Suzuki et al. 2019). However, perceptions of (or inferences about) causality itself are a basic component of sense of agency (e.g. Kawabe et al. 2013). While intentional binding should not be considered as a diagnostic marker of the presence or absence of agency, its can still be treated as a comparative proxy for it (i.e. allowing to compare conditions differing in the degree to which they may modulate agency; Wen and Imamizu 2022). Further, comparing temporal binding between a voluntary movement condition and a relevant control condition such as passive movement can effectively isolate the specific component of binding associated with intentional action.

Here, we measured intentional binding between a ballistic thumb movement (the action) that caused an auditory tone (the outcome), both before and after motor training in two groups. One group trained on the same thumb movement used in testing, the *relevant training* group. The other group trained on a different movement to that tested and were, therefore, designated an *irrelevant training* group.

Theories of action control make contrasting predictions about the effects of motor skill learning on sense of agency. On the one hand, a dominant theme in motor learning is automatization. Evidence from various domains of skill acquisition, such as elite sports performance, suggests that performance requires less attention with increasing practice (reviewed in Fitts and Posner 1967). For example, Schaefer and Scornaienchi (2001) showed expert table tennis players experienced 10% costs to performance from a dual-task while returning balls, compared to novices who experienced 30–50% costs. Relatedly, elite air pistol shooters show a global drop in cortical activity during shooting, which may reflect neural efficiency and spatially selective processing (Del Perico et al. 2009; also see Debaere et al. 2004; Puttemans et al. 2005). Given well-learned actions involve a less intentional, less cognitive form of control than a novel action, sense of agency might be expected to reduce with motor learning. On the other hand, previous studies of action–outcome learning suggested that fluency is associated with strong agency ratings (Wenke et al. 2010; Chambon and Haggard 2012). However, those studies focussed on fluency of selecting between multiple actions, rather than skill in executing a single action. Here, we predicted reduced intentional binding over the trained thumb movement for the relevant training group (compared to the irrelevant training group) after training.

In this study, we did not examine binding of the action towards the tone (‘action binding’). This is because our training intervention aimed to change action performance, meaning we would risk comparing binding for actions that were not physically identical. Rather, we focussed on the binding of the tone towards the action that caused it (‘tone binding’). Because the tone remains constant throughout the experiment, any tone binding changes would reflect a psychological consequence of motor learning on our measure of sense of agency.

## Methods

### Participants

There were 19 participants in the relevant training group (age, *M* = 30.52, SEM = 2.45; 7 males; 2 left-handed), and 18 in the irrelevant training group (*N* = 19, 1 outlier subsequently excluded; age, *M* = 28.73, SE = 1.90; 10 males; 1 left-handed). Group allocation was random. All participants provided informed consent, and ethical approval for the study was granted by the Institute of Cognitive Neuroscience Ethics Chair, University College London (approval number: ICN-PH-PWB-20–02-2014c).

### General procedure

**Fig. 1:**
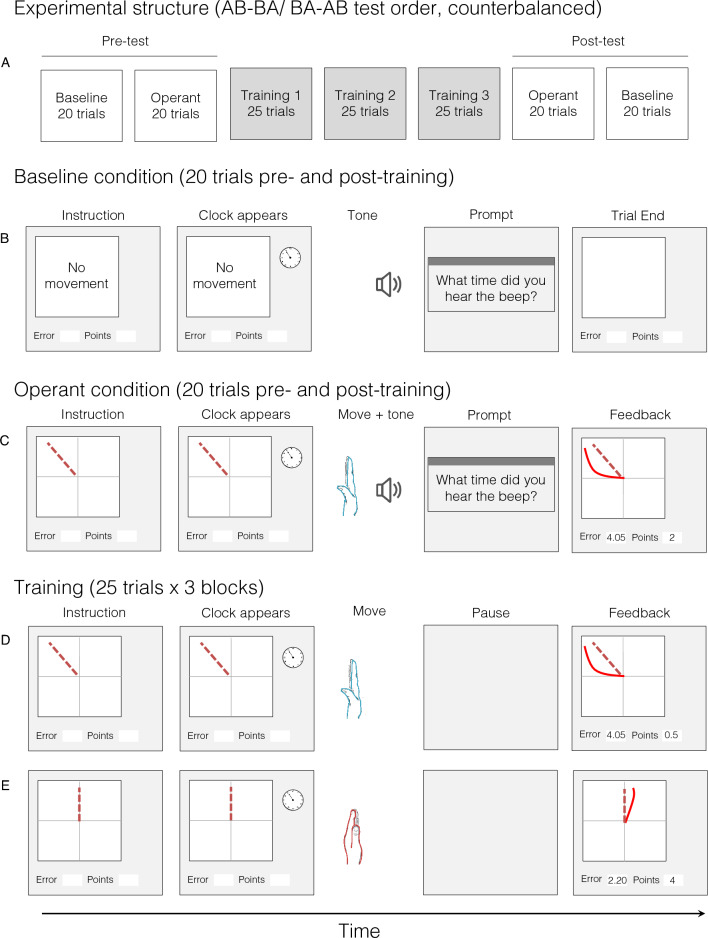
Trial structure for different blocks/conditions and visual feedback presented: **A** experimental structure: the pre- and post-tests consisted of baseline and operant blocks, counterbalanced in an AB-BA or BA-AB order. Pre- and post-tests were identical between participants. Participants then completed training, with the trained movement depending on group assignment. **B** Baseline block structure: participants saw a ‘no movement’ instruction, the clock appeared, and the tone sounded after a random delay. A prompt box appeared, and participants made their judgement of the time of the auditory tone (no movement was made); **C** operant block structure: participants saw an instruction image cueing the movement to be performed (up-left movement), the clock appeared, participants performed the movement at a time of their choosing. This movement triggered the tone. After a random delay, the clock disappeared, and participants were prompted to fill in the time of the tone. They then received feedback on their movement performance (solid line) versus the target movement (dashed line), as well as an error score and points. **D** Training block, relevant training group: the movement instruction appeared, then the clock appeared, and the participants made the up-left movement (same movement as in the pre- and post-tests). No tone was presented, and thus, no timing judgement required. **E** Training block, irrelevant training group: identical procedure to training performed by the relevant-group training, though with a different (upwards) movement performed

Participants first completed a short (3–5 trials) familiarisation on how to perform the tested movement. Next, all participants completed a pre-test, consisting of a baseline block and an operant block (20 trials each, order counterbalanced; more details below). Both the familiarisation and pre-test were identical between groups (see Fig. 1 for timeline). After the pre-test, both groups underwent training (3 blocks of 25 trials, ~ 1–3-min breaks between blocks). Finally, both groups completed the post-test. This post-test was identical to the pre-test, and common to both groups.

### Baseline trial procedure

On baseline trials, an auditory tone was randomly triggered 3–5 s after the trial began. Participants judged the time of this tone by reporting the position of a rotating clock hand, using mental chronometry methods reported previously (Libet et al. 1983). The actual time of the tone was subtracted from the reported time to calculate a judgement error (i.e. error = reported − actual). Thus, positive values indicate the tone was reported to occur after it occurred in reality, implying a perceptual lag.

### Operant trial procedure

In operant trials, an instruction image appeared representing the movement to be performed. The clock then appeared after a random delay indicating that participants were free to make the thumb movement at a time of their choosing. Thumb movements began from a neutral starting position: the hand rested on its ulnar aspect on a foam support with the thumb facing upwards, aligned with the midline. Participants performed a fast (~ 1 s), upwards-leftwards movement of the thumb (see Fig. 1). Movement was recorded using a 2-axis accelerometer (see Supplementary Materials for recording and analysis information). Ballistic aimed movements of the thumb have been widely used as a paradigm of motor skill learning (Classen et al. 1998; Liepert et al. 1999; Butefisch et al. 2000).

Moving the thumb triggered the auditory tone 250 ms later, and participants judged the tone time as above. Next, a 2D visual representation of movement performance (on that trial) versus the instructed ‘ideal’ movement was presented (see Fig. 1). They also received a movement ‘error’ value representing the deviation of their movement from the ideal movement (see Supplementary Materials for calculation details), and points based on this error. Points were for motivational purposes and to calculate payment bonuses.

### Calculating intentional binding

As our main dependent variable, an intentional binding score was created by subtracting average baseline from operant timing judgements. Negative binding scores indicate a shift in tone judgement towards the time of the thumb movement that triggered it, consistent with an intentional binding effect.

### Training procedure

The *relevant training* group trained on the same up-left movement used in the pre- and post-testing blocks. In contrast, the *irrelevant training* group trained on a different movement (also ~ 1 s, from the same starting position). This movement was different in that, rather than moving the thumb up and leftward (as with the tested movement), participants had to move the thumb directly up in the air. Before training, participants in the irrelevant training group received a short familiarisation (also 3–5 trials) on how to perform this new movement. Training trials were identical to operant trials (see Fig. 1), though participants were not required to make any chronometric judgements to allow them to focus on movement performance. Consequently, no auditory tones were presented during training. Protocols for calculating error and awarding points were also identical to operant trials.

Participants were not aware of what movement they were performing for training, or that this might be different to the operant test, until after the pre-test. Previous studies (Classen et al. 1998) showed a very high degree of directional specificity of the motor plasticity underlying learning of such thumb movements, making us confident that any learning in the irrelevant training group would be unlikely to transfer to the test movement direction. Further, any such learning transfer in the irrelevant training group would count against the hypothesised difference between the groups.

## Results

### Manipulation check: motor error

We first validated our training intervention with a manipulation check examining motor performance using the error scores described above. We expected post-test movement error to be significantly lower (indicating better performance) for the relevant training group compared to the irrelevant training group because the relevant training group received training on the test movement.

An unexpected difference was identified in movement error between groups at pre-test, with the relevant-practice group showing more error than the irrelevant-practice group despite them both performing the same test movement (*t* (35) = 2.45, *p* = 0.019, *d* = 0.81; see Table 1A).

**Table 1:** Movement error results I—testing

Movement error results I—testing		
Comparison	**A.** Independent samples t-test: DV pre-test error; factor Group (relevant vs. irrelevant training)	**B.** ANCOVA: DV post-test error; factors Group (relevant vs. irrelevant training), and pre-test error (covariate)
Group	*t* (35) = 2.45, ***p* = 0.019**,* d* = 0.81	*F* (1,34) = 5.08, ***p* = 0.031**,* η*_p_^2^ = 0.13
(Covariate) Pre-test move. error	NA	*F* (1,34) = 30.94, *p* < 0.001,* η*_p_^2^ = 0.47

**Fig. 2:**
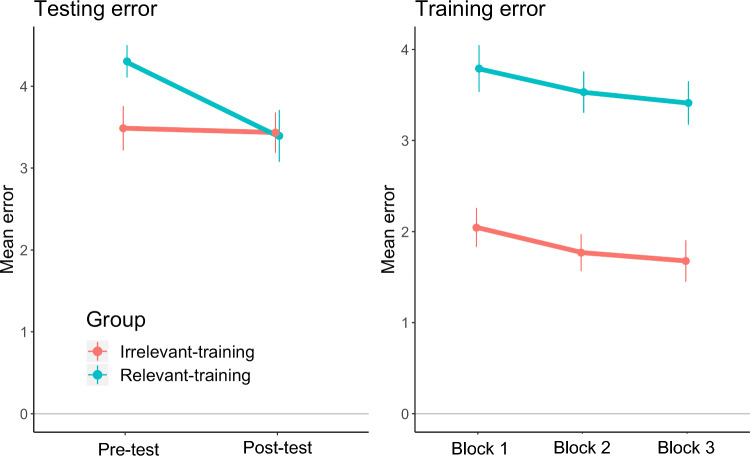
Movement error data during testing (left panel) and training (right panel). Left panel: during testing, the relevant training group (blue line) improved in performance of the ‘up-left’ thumb movement (i.e. from pre-to post-test), where the irrelevant training group (red line) did not. When accounting for the pre-test error difference between groups, the relevant training group has significantly lower error than the irrelevant training group at post-test (see Table 1B). Right panel: while either group trained at performing a different movement (‘up-left’ versus ‘upward’ for the relevant and irrelevant groups, respectively) both groups showed a similar amount and rate of learning from the first to last training block. Please note, drops in motor error indicate improved motor performance. Error bars are standard error of the mean. To see these values represented as change scores (pre-post), please see Supplementary Fig. S1

**A** There was an error difference between groups at pre-test, with more error for the relevant training group; **B** error at post-test was also significantly different between groups, but in the opposite direction: more error was seen for the irrelevant training group (when accounting for pre-test error with ANCOVA). *Note*: lower error means better performance of the thumb movement. See Fig. 2 (left panel) for visual representation of these results. *d* values represent Cohen’s *d*. All *t*-tests are two-tailed, *α* = 0.05

Thus, for subsequent comparisons, the influence of pre-test error was removed by analysis of covariance (ANCOVA). ANCOVA has several statistical advantages for studying pre-test/post-test designs, relative to more familiar ANOVA of change scores, and is recommended in situations where groups differ at baseline, as here. On the basis of a comprehensive simulation study, Egbewale et al. (2014) concluded: “When baseline imbalance is in the opposite direction from the treatment effect, ANCOVA corrects the resulting bias by producing an adjusted treatment effect that is larger than the nominal treatment effect, and ANCOVA therefore has greater power to detect this effect than ANOVA has to detect the nominal effect, at the same sample size” (Egbewale et al. 2014; page 9). In the current study, the baseline imbalance meets this criterion, being in the opposite direction to our expected intervention effect (more movement error at baseline in the relevant training group; the group expected to have lower error after experiencing training relevant to the post-test measure. For further discussion, see the Supplementary Materials.

A mixed ANCOVA with pre-test error as a covariate demonstrated that, as expected, the relevant training group was better at performing the up-left thumb movement at post-test, compared to the irrelevant training group (who trained on a different movement). This was reflected in significantly lower error for the relevant training group (*F* (1,34) = 5.08, *p* = 0.031, *η*_p_^2^ = 0.13; see Fig. 2 (left panel) and Table 1B). Thus, while the relevant training group was worse than the irrelevant training group at pre-test, they were significantly better at post-test (when accounting for this pre-test difference).

This between-group difference was supported by within-group comparisons. The relevant training group showed significant improvement in error from pre- to post-test (*t* (18) = 3.90, *p* = 0.001, *d* = 0.90), but there was no significant change between tests for the irrelevant training group (*t* (17) = 0.30, *p* = 0.770, *d* = 0.07); please see Fig. 2 (left panel). In sum, our manipulation check demonstrated the training intervention was indeed successful in producing significant learning on the tested movement for the relevant training group only.

While not of direct experimental interest, we also looked at motor error during training. The groups were trained on different movements (up-left versus upward, for the relevant- and irrelevant training groups, respectively), but we expected significant improvements in both groups and of similar magnitudes because pilot testing indicated the movements were of similar difficulty.

A mixed ANOVA demonstrated both groups showed a drop in error for their specific trained movement over blocks (*F* (2,70) = 4.74, *p* = 0.012, *η*_p_^2^ = 0.12), and that this drop was consistent between groups (no interaction; *F* (1,70) = 0.01, *p* = 0.994, *η*_p_^2^ = 0.01); see Table 2 and Fig. 2 (right panel). As with error at the operant pre-test, there was increased error overall for the relevant training group during training (main effect of group; see Table 2). This was not considered problematic as both groups showed learning from training. Critically, only the relevant-practice group learned on the *testing* task from pre- to post-test.

**Table 2:** Movement error results II—training

Movement error results II—training	
Comparison	**D.** Mixed ANOVA: DV training error; factors Block (block 1–3), Group (relevant- vs. irrelevant training)
Block	*F* (2,70) = 4.74, ***p* = 0.012**, *η*_p_^2^ = 0.12
Group	*F* (1,35) = 35.82, ***p* < 0.001**, *η*_p_^2^ = 0.51
Block × Group	*F* (1,70) = 0.01, *p* = 0.994, *η*_p_^2^ = 0.01

Both groups showed a drop in error that was of the same rate. A group difference was identified where the relevant training group showed more error overall, consistent with the difference seen in pre-test error between groups. See Fig. 2 (right panel) for visual representation of these results

### Experimental comparisons: intentional binding

Our main interest focussed on intentional binding measures. Binding values were not significantly different between groups at pre-test (*t* (35) = 0.76, *p* = 0.454, *d* = 0.25; see Table 3A). However, to account for any non-significant differences at pre-test, and for consistency with the analysis of movement error, we used ANCOVA to remove differences in pre-test while comparing the post-test across groups (Table 3B).

**Table 3:** Intentional binding results I—testing (between groups)

Intentional binding results I—testing (between groups)		
Comparison	**A.** Independent samples *t*-test: DV pre-test binding; factor Group (relevant vs. irrelevant training)	**B.** ANCOVA: DV post-test binding; factors Group (relevant- vs. irrelevant training), and Pre-test binding (covariate)
Group	*t* (35) = 0.76, *p* = 0.454,* d* = 0.25	*F* (1,34) = 5.22, ***p* = 0.029**,* η*_p_^2^ = 0.13
(Covariate) Pre-test binding	NA	*F* (1,34) = 12.16, ***p* < 0.001**,* η*_p_^2^ = 0.26

**A** Pre-test binding was not significantly different between groups at pre-test. **B** At post-test (after the training intervention), however, a significant difference in binding appeared. Specifically, the relevant training group now showed significantly less binding compared to the irrelevant training group. *d* values represent Cohen’s *d*. All t-tests are two-tailed, *α* = 0.05

**Fig. 3:**
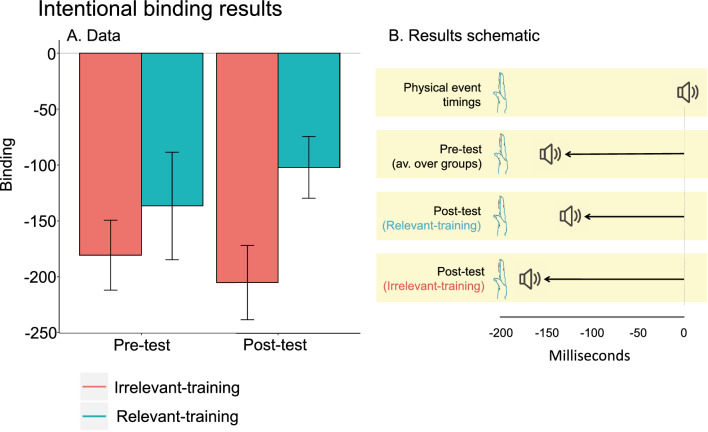
Intentional binding results and schematic summary. **A** While both groups demonstrated intentional binding at pre- and post-tests (indicated by negative values), at post-test, binding was significantly reduced (less negative) for the relevant training group relative to the irrelevant training group. Error bars are standard error of the mean. **B** A schematic of how intentional binding changed over tests. Less binding at post-test means the perceived time of the tone is less shifted towards the thumb movement, as seen in the relevant training group. For the irrelevant training group, in comparison, the tone shifted slightly more towards the movement time

A mixed ANCOVA with pre-test binding as the covariate, demonstrated that there was a significant difference between groups at post-test (*F* (1,34) = 5.22, *p* = 0.029,*η*_p_^2^ = 0.13; see Table 2B). Thus, after the training intervention, there was less binding in the relevant training than in the irrelevant training group (see Fig. 3). As expected, this binding group difference was the result of changes in the operant but not baseline conditions following training (see Supplementary Table S1).

Descriptively, binding scores reduced slightly for the relevant training group pre- to post-test and increased slightly for the irrelevant training group (see Fig. 3), leading to the post-test group difference we identify. Neither of these pre-to-post changes were significant when considered on their own with repeated-measures *t*-tests (relevant training group, *t* (18) = − 0.86, *p* = 0.399, *d* = 0.20; irrelevant training group, *t* (17) = 0.76, *p* = 0.457, *d* = 0.17). While this limits the conclusions that can be drawn from this study, our key inference, that training influences intentional binding, is based on the group main effect in ANCOVA, and does not require that the experience-dependent change in either group be significant of itself. Indeed, both groups showed significant binding both at pre- and post-tests. This was shown by negative mean binding scores for both groups, both of which were significantly below than zero (both *p* ≤ 0.011; see (see Table 4A and B). Therefore, binding was changed but not eliminated by the training intervention in either group.

**Table 4:** Intentional binding results I—testing (presence of binding)

Intentional binding results I—testing (presence of binding)		
	Pre-test	Post-test
Comparison	**A.** One sample *t*-test: DV pre-test binding	**B.** One sample t-test: DV post-test binding
Relevant training group	*t* (18) = − 2.84, ***p* = 0.011**,* d* = 0.65	*t* (18) = − 3.70, ***p* = 0.002**,* d* = 0.85
Irrelevant training group	*t* (17) = − 5.75, ***p* < 0.001**,* d* = 1.36	*t* (17) = − 6.17, ***p* < 0.001**,* d* = 1.45

**A** As expected, at pre-test, the intentional binding effect was seen in both groups, i.e. significant negative values. **B** At post-test, both groups still showed significant intentional binding, indicating training did not abolish binding, but rather produced a greater reduction in binding in the relevant training compared to the irrelevant training group. *d* values represent Cohen’s *d*. All *t*-tests are two-tailed, *α* = 0.05

## Discussion

In sum, we successfully trained the relevant training group to have significantly improved performance of the tested thumb movement compared to the irrelevant-practice group. After relevant training, intentional binding between action and outcome was reduced, relative to a group given irrelevant training, and after adjusting for the pre-test level of binding using ANCOVA. This indicates there was relatively less binding between a volitional action and its outcomes when the action had been practised to an increased level of skill. How could a training-based change in motor processing link with a change in the subjective experience of agency, as reflected by changes in intentional binding? This can be understood by first considering how motor processing changes with practice.

Previous research has demonstrated decreases in brain activity, particularly in prefrontal, premotor, parietal and cingulate areas, during motor skill acquisition (Debaere et al. 2004; Puttemans et al. 2005). These changes have been suggested to reflect reductions in attention-demanding sensory processing, as well as the suppression of motor tendencies unhelpful for skilled performance (Puttemans et al. 2005). Indeed, as learning progresses towards automatisation, performance is increasingly driven by feedforward rather than feedback control (Wolpert et al. 1998; Debaere et al. 2004). This switch may allow attention to be freed up for allocation to other processes. Indeed, directing conscious attention to a highly automatised movement has, in fact, been shown to disrupt skilled movement (Wulf et al. 2001; see Beilock and Carr (2001) for evidence of the link between explicit monitoring and ‘choking’ in expert golfers).

In the current experiment, we used a simple, ballistic movement that was easily learned, in contrast to more complex tasks which require days to achieve a high level of automaticity (e.g. Puttemans et al. 2005). Thus, extended practice for the relevant training group presumably led to a relative reduction in the processing of sensorimotor feedback resulting from the thumb movement as it progressed towards automatization, as compared to the irrelevant training group.

Computational models of motor control suggest the sense of agency is based on the comparison of predictions and sensory evidence (Blakemore et al. 2002). In the relevant training group, movement training may have led to the sensory feedback from the tone being better predicted relative to both the irrelevant training group and the pre-test condition. Interestingly, this improved prediction of the sensory consequences of movement would be an incidental by-product of training rather than a direct product of training, because the sound did not contain any information about the trained variable, or movement direction. Nevertheless, the effects of training might generalise, to encompass both prediction of the visual feedback used to indicate movement direction, and prediction of the incidental tone that followed each movement. Sensory feedback from the tone might, therefore, be largely cancelled out. After relevant training, any prediction error signal caused by the action-evoked tone would be minimal. Interestingly, the intentional binding measure of sense of agency reduced with training, as prediction presumably also improved, and prediction error correspondingly decreased.

Our finding of a drop in intentional binding with practice is consistent with other agency research. In a previous study by Morioka et al. (2018), participants all performed the same task and were later split into post hoc groups based on how much learning they demonstrated. Morioka et al. were unable to find a group difference in intentional binding between the two groups, but they do report the higher learning group decreased in intentional binding, though between task blocks two and five only. No changes were seen for the low learning group. We randomly allocated participants in advance to two different training groups, which provides a stronger experimental design than relying on individual differences in learning. Both studies, however, converge in their suggestion the sense of agency, as measured by intentional binding, reduces with perceptual-motor learning.

One alternative account of observed intentional binding results regards potential differences in the rewarding value of the task. It is possible that improvement in the tested movement could have led to more satisfaction and reward expectations in the relevant training group, compared to the irrelevant group, which did not show such an improvement. In such a view, a difference in binding at post-test between the groups could be driven by these subjective factors, rather than occurring as a function of movement error. However, the evidence for the relationship between the rewarding value or the valence of action outcomes and intentional binding is mixed. Some studies reported reduced intentional binding following negative outcomes (Takahata et al. 2012; Yoshie and Haggard 2013), some suggested no such influence (Moreton et al. 2017), and yet others showed that losses can lead to intentional binding enhancement (post-error agency boost; Di Costa et al. 2017; Majchrowicz et al. 2020). Unclear direction of such potential effect, and the lack of direct probing of these aspects of subjective experiences of our participants, leaves this issue out of scope of this work.

Reductions in frontal activity reported to occur alongside motor practice (Debaere et al. 2004; Puttemans et al. 2005, see above) may directly reflect the reduction in networks related to the conscious intention for action—and thus associate with the drops in intentional binding we report here. Indeed, frontal brain regions, particularly the pre-supplementary motor area (pre-SMA), play a critical role in conscious intentions for voluntary action. This is reflected by the increase in electrical activity over the pre-SMA just prior to the initiation of internally generated actions (the ‘readiness potential’: Libet et al. 1983; Yazawa et al. 2000). Evidence also comes from direct stimulation of the pre-SMA in awake humans producing the subjective ‘urge’ for movement, with stronger stimulation producing execution of the movement for which patients experienced an ‘urge’ at lower stimulation levels (Fried et al. 1991; also see Jenkins et al. 2000 regarding the role of rostral SMA in self-initiated movement).

In conclusion, here we provide evidence that implicit measures of agency over an action reduce as that action becomes more practised. While interesting, this result should be considered preliminary. While we were able to demonstrate a skill acquisition-related difference in intentional binding between our training groups (our comparison of interest), the absolute change in binding within each group from pre- to post-test was not significant. Further, while we statistically accounted for the initial difference in movement error between groups, future research should investigate whether initial error rates might affect changes in intentional binding. Despite these limitations, our results provide initial support for a drop in agency related to a movement with practice, which may indicate decreasing cognitive engagement or automatisation that occurs during skill learning.

## Supplementary Information

Below is the link to the electronic supplementary material.

Supplementary file1 (DOCX 857 KB)

Supplementary file2 (XLSX 13 KB)

## Data Availability

All data generated or analysed during this study are included in this published article and its supplementary information files. See supplementary Excel file.
