# Supplementary material for: Implicit signatures of voluntary action reduce with repeated motor practice
Source: Exp Brain Res. 2023 Aug 24;241(9):2361–70. doi: 10.1007/s00221-023-06675-w (PMC10471669; doi:10.1007/s00221-023-06675-w)
Supplement: Supplementary file 1 — Supplementary file1 (DOCX 857 KB) [file 221_2023_6675_MOESM1_ESM.docx]

**Title:**

Implicit signatures of voluntary action reduce with repeated motor practice

**Authors:**

Dempsey-Jones, Harriet^1,3^, Majchrowicz, Bartosz^2^ & Haggard, Patrick^1^

**Affiliations:**

^1^ Institute of Cognitive Neuroscience, University College London, United Kingdom

^2^ Institute of Psychology, Jagiellonian University, Krakow, Poland

^3^ School of Psychology, University of Queensland, Australia

**Corresponding author:**

Patrick Haggard, [p.haggard@ucl.ac.uk](mailto:p.haggard@ucl.ac.uk) (ORCiD: 0000-0001-7798-793X)

**Journal:**

Experimental Brain Research

**Supplementary Materials**

**Supplementary Methods**

*Participants*

Participants were randomly allocated into one of two groups based on order of recruitment. One participant was excluded from the irrelevant training group due to outlying data (more below), leaving 18 participants.

*General procedure*

All participants completed a pre-test, training and post-test procedure – in that order. The pre- and post-tests consisted of a block of baseline (tone only) timing judgments (20 trials), and a block of operant (tone and movement) judgments (20 trials). Baseline and operant judgments were counterbalanced in an AB-BA order between pre- and post-test, with half the participants starting with the baseline condition, and half with the operant condition (see Figure 1 in the main text). Pre- and post-tests were identical between groups, and are described in more detail below.

All participants completed a training block, with the type of training varying depending on group assignment. For the relevant training group, the type of movement trained in the training block was identical to that performed in the operant pre- and post-test blocks (in both cases, an up-and-left thumb movement, more below). For the irrelevant training group, participants trained on a different movement (upwards thumb movement) to that they performed in the operant pre- and post-tests (up-and-left thumb movement, identical to that performed in training and testing in the relevant training group). Thus, testing movements were comparable between groups, but the irrelevant training group performed an alternate movement during training. Thus, any differences between groups on the post-test measures would be attributable to one group having trained on the testing movement (and any associated learning), where the other group did not.

In all conditions using a clock for timing judgments the details of the clock were consistent, and as follows: the clock was numbered from one to 60 in increments of 5. It was 15 millimetres (mm) in diameter on screen – where the screen was ~600mm from the seated participant.

When clock appeared on screen, the hand on the clock would rotate at 1 revolution/ 2560 milliseconds (ms). The clock hand would continue to rotate until a condition was met (which varied depending on the task, see below). Following the condition being met, the clock hand would continue to rotate for a further interval (between 3,000 and 4,000ms, randomly determined trial-by-trial), and then disappear from the screen. A dialogue box would then pop up prompting participants to indicate the time on the clock at which the condition was met (the actual condition also varied by task, e.g., when you heard the tone; more below). Participants used a keypad to enter the clock time from 1-60, and then pressed Enter to continue.

All participants completed a short familiarisation and training of each block type (baseline, operant, training) prior to commencing the block. The familiarisation sessions all consisted of 4 trials of the relevant type with instruction and coaching from the experimenter (following a standard script), conduced immediately prior to commencement of the first experimental block.

*Baseline (tone only) test*

The baseline test was administered at pre- and post-test. It allows a measure of participant’s estimation of the timing of a tone alone (when this tone is triggered randomly, not by a preceding movement).

As mentioned above, there were 20 trials in each baseline block. On a single trial, the instruction would appear on screen, after 2000ms the clock would also appear on-screen and start rotating, there would then be a short interval (1000-3000ms, randomly determined), followed by a 250ms tone. The clock hand would continue to rotate for another random interval (3000-4000ms, randomly determined), before disappearing. The dialogue box would then pop up, prompting participants to make their time judgment (‘What time did you hear the tone?’).

An independent measures t-test indicated there were no significant differences between the relevant training and irrelevant training groups in this baseline time estimation at pre-test (t(35) = -1.20, p = .239), indicating consistency between groups prior to the intervention.

*Operant (movement and tone) test*

The operant test was also administered at pre- and post-test. This test looks at how participants judge the timing of a tone that was initiated by a movement of their thumb.

There were also 20 trials of this test per operant block. On a single trial, participants would place their hand in the starting position (as demonstrated by the experimenter in the operant familiarisation session). The starting position consisted of participants holding their hand on its side with fingers together (little finger down), resting on a foam support, approximately oriented with their midline.

The first trial would begin with an ‘instruction’ image appearing on-screen. This image showed a schematic representation of the movement they were about to perform (which was the same each time, within a block). Figure 1 in the main text shows an example of this instruction image: this consisted of a black background overlaid with a 2D plane – with *x* and *y* axes indicated by white solid lines. At an approximately 315° angle from upright (0°) there was a white dotted line, indicating the participant needed to make a thumb movement in the up-and-leftward direction from the starting position of approximately the same angle from the mediolateral plane.

This instruction would stay on screen for 2000ms. When this disappeared, the clock would appear on screen and rotate until participants completed the up-left thumb movement (more below). When the participant’s movement exceeded a set threshold (.5g, more below) this would trigger a tone to sound after a 250ms interval (same tone used in the baseline tests).

After the tone, the clock hand would continue to rotate for a following interval (3000-4000ms, randomly determined). The clock would then disappear, and the dialogue box would appear prompting participants to make their judgement (‘What time did you hear the tone?’). Participants would enter the time using a keypad, and then press enter when they were ready to commence the next trial.

After the first trial, the participant’s performance, in terms of position change of the thumb, was overlaid as a red line onto the instruction screen. Just underneath the instruction screen, participants were given an ‘error’ number, which indicated how far their movement deviated from the up-left target movement of the operant block. Participants were also given a number corresponding to the ‘points’ they had won for that performance on that trial, and this was added to a running total score – both also presented underneath the instruction/ feedback image on screen at all times. Details of error calculations and points assignment are outlined in full below.

This visual feedback was included, and left on screen between trials in order to allow participants to best learn from their performance. Error and points were included to sustain attention and motivation – participants were informed they were able to gain up a nominal amount of financial reward for good performance (up to 50p, based on points gained in testing and training – though participants were not informed further on the schedule for bonus payment).

An independent measures t-test indicated there were no significant differences between the relevant training and irrelevant training groups in this operant time estimation at pre-test (t(35) = 0.24, p = .814), indicating consistency between groups prior to the intervention.

*Training task*

In the training task, the relevant training group performed the same up-left movement as they did in the operant testing task. The irrelevant training group performed a new movement where the thumb would move directly upwards from the hand starting point (which was the same as for the operant test). That is, the movement required the thumb to move at 0°, upwards from its starting position (see Figure X). Both groups performed a short familiarisation (procedure as above) before commencing the first block of training.

Participants were not informed until the start of the training session that they would be performing the same/ different movement in the training, and were thus, blinded to this manipulation during the pre-test.

The training trials were similar to the operant testing trials, with the exclusion of the tone following the movement, and thus, the requirement to judge the timing of said tone (and no dialogue box for recording said response). In sum, participants first placed their hand in the starting position, the instruction/ instruction & feedback image appeared (depending on whether it was the first or subsequent trials, respectively), they performed the required movement, and then received feedback on their performance (visually, as well as through error and points values). All other timings and details remained consistent with the operant condition.

*Movement error and points calculations*

Movement was recorded using an accelerometer (ADXL355, Analog Devices, Norwood, MA: [www.analog.com](http://www.analog.com)), linked to a National Instruments box, and a laptop computer running LabVIEW version 2017 with NI-DAQ toolboxes installed (National Instruments, Berkshire, UK: <http://www.ni.com>).

The accelerometer was mounted on the dorsum of the thumb – between the end of the nail and the most distal thumb joint. Two dimensions of acceleration were recorded (corresponding with proximo-distal – towards and away from the body, and medio-lateral – from left to right in a perpendicular direction to the first axis). This was due to the stationary position of the hand, and the overall restriction of movement in the third dimension due to the anatomy of the hand.

Details for NI-DAQ instrument initialisation were as follows: 2000 samples/ sec (continuous sampling mode; buffer size 5000), min/ max volts set to 0/3.5, input is referenced to ground: RSE, sample clock source was the on-board clock. The two channels of voltage input from the accelerometer were corrected for biases in voltage at zero-g (by removing baseline voltage biases; using recommendations from product guidelines, and comparing with zero-g recordings; done separately for either accelerometer). Voltage was then converted to g (g_to_ = voltage_to_/ (325/1000); where t0 is the reading of voltage at the current time). A Savitzky Golay filter (6 side points; 3^rd^ order polynomial) was used to smooth the g data. This was done to improve data quality, and to prevent noise/ unintentional movements from triggering the tone.

As stated above, in the operant condition, movement above a certain threshold would trigger the tone. A moving window (CT_-1000_:CT: where CT is current trial reading) was used to determine whether sufficient movement had occurred to trigger the tone. If the mean g within this window exceeded .5g the trigger occurred.

To convert the acceleration values into position information, we first used the baseline values to correct for initial position of the thumb to determine direction of acceleration. To do so, we subtracted the mean baseline g value (collected over the 2000ms no movement baseline period where participants kept their hand completely still, and waiting for the clock to appear; see above) from movement trial values. We created a vector representing the movement of the thumb from the starting position out to the end of its movement trajectory (cutting before the thumb began to return to its starting position). This was done by cutting the movement vector from the start of the movement that triggered the tone, to the point where the acceleration reversed (as the thumb moved back to its original position). The acceleration was then converted to position information by a double integration (dt: 0.0005; trapezoidal integration).

The target movement (either ‘up-left’ or ‘up’, depending on trial type and condition) was represented by a vector of coordinates corresponding with 2D position change over time, i.e., how the thumb changed position over the time it was moving. To assess correspondence of the participant’s movement with the target movement on any given trial, this vector of position change coordinates was compared with the vector representing the participant’s movement. To compare position, the end-point and the mid-point of the target and actual movement were taken (for x and y axes, separately). The actual end-point was subtracted from the target end-point to determine the difference in both the *x* and *y* dimensions, and the absolute value was taken (see formula_1_ below). This was repeated for the mid-point error. Mid- and end-point error was then summed for the x and y values to produce a total error value (see forumula2 below).

Formula_1_: error_xMid_ = | target_xMid_ – actual_xMid_ | x 1000

Formula_2_: error_total_ = error_xMid_ + error_yMid_ + error_xEnd_ + error_yEnd_

Mid-, as well as end-point error was taken to represent when there was a deviation in the progression of the thumb to its end point, e.g., a curved movement. The absolute deviations for the mid- and end-points of the target vs. actual movement were then added together to give a single number representing ‘error’ – with 0 representing no difference between the target and actual line at the two measurement points, and larger numbers indicating worse performance.

Points were calculated for each trial based on error: 8 points were assigned for total error scores between 0-0.5, 6 points for 0.5-1, 4 points for 1-2, 2 points for 2-3, 1 point for 3-4, and 0.5 points for 4-5, and 0 points for error above 5.

*Pre-processing of tone time judgements*

All judgements of the tone timing using the clock (in the baseline and operant tests) for each trial were pre-processed prior to analysis. First, all time values were converted from 1-60 clock units (as displayed to participants on screen) to millisecond format, using the following formula: T_ms_ = T_clock_ x (2560/60), i.e., as 1 clock rotation took 2560ms.

A difference score was then calculated to represent the difference in the participant’s reported time of the tone and the actual time of the tone using the following formula: T_response_ – T_actual_. This was done separately for each trial/block/participant. This calculation, therefore, produced positive value if participants reported the tone occurred *after* its actual time (a delay) and negative values if they reported a tone time that occurred *before* the tone actually occurred (an anticipation).

A small adjustment was required in the situation where a participant reported a value that fell in a different rotation of the clock to the time the tone actually occurred, e.g., if the tone occurred at 59 on the clock, but the participant reported a 1 (as the clock hand had passed 60, and entered a new rotation). A standard rule was applied to deal with causes such as this – reports that were over half the full rotation of the clock forward in time from the tone, i.e., over +1280ms (+30 clock units) were subtracted from 2560ms (60 clock units). Consistently, values that were over half a rotation in the opposite direction, i.e., below -1280, had -2560 subtracted from that value. As stated above, the same rule was applied to all participants for comparability between groups. The average instance of such extreme values was low, on average ~7 trials from 80/ participant (over all conditions at pre- and post-test). Please note, the general picture of results was preserved without this correction.

Formula_3_: If time <= -1280, time_new_ = time – -2560

e.g., time = -2517, time_new_ = 2517 – -2560

time_new_ = 42ms

Formula_4_: If time >= +1280, time_new_ = 2560 – time

e.g., time = +2517, time_new_ = 2560 – 2517
 time_new_ = -42ms = -1 clock units

*Calculating ‘intentional binding’ outcome measure*

Finally, we constructed an intentional binding value for each participant, to represent how much the estimated time of the tone changed when the tone was presented on its own (baseline test), as compared to when it was trigged by/ followed a thumb movement (operant test).

To calculate this, an average for the baseline test was computed for each participant, and an average for the operant condition. For each participant, the average baseline value was subtracted from the operant value to create the intentional binding score.

As discussed in the Introduction, intentional binding is said to occur when judgment of the time of an event is dragged from its baseline position (when the event occurs on its own), towards the time of a preceding movement (Haggard et al., 2002; Moore & Obhi, 2011; reviewed in Haggard et al., 2005; 2008). That is, the time of the tone shifts earlier in time, and is reported by the participant to have happened closer to the action that triggered the event.

Thus, negative intentional binding (binding) values indicate a shift of the tone judgment towards the action time (earlier in time) – consistent with results from previous studies. This means that, if the intentional binding effect got larger between pre- and post-training measures, it would become a more negative value, e.g., from -5ms to -10ms. Positive binding values indicate the operant tone judgment occurs after the baseline judgment (later in time) – which would be in the opposite direction to that predicted. A reduction in intentional binding from pre- to post-test would be reflected in scores that become more positive, e.g., from -5ms to -2ms.

*Use of ANCOVA vs. ANOVA methods in our analyses*

There are several issues that can be caused by imbalances in baseline measures (Egbewale et al. 2014; Clifton & Clifton, 2019). The most obvious of these is that if two groups have different pre-test scores, you cannot easily compare post-test scores.

One approach to dealing with this is change analysis: where the pre-test value is subtracted from the post-test value (change = post-pre), to create difference scores, that are then compared between groups.

Unfortunately, there are several issues that these methods cannot resolve (Clifton & Clifton, 2019), like regression towards the mean (Galton, 1886). In our study, for example, because the relevant-training group starts off with error values that are significantly greater (more extreme) compared to the irrelevant-training group, it is likely that a greater drop in error would be seen for the relevant-training group just under repeated testing alone (i.e., with no intervention). This is because the scores are more extreme in this group at pre-test, making it more likely the values will naturally drop towards the mean at the post-test (Galton, 1886).

Analysis of Covariance (ANCOVA) methods address these problems. Indeed, ANCOVA’s regression-based approach has been shown to produce unbiased estimates of intervention effects in the face of baseline imbalances when group assignment is randomised (Huitema, 2011).

Egbewale et al. (2014) used simulations to examine how both baseline imbalances and pre-post correlations affect ANOVA and change analysis methods, as compared to an ANCOVA approach. They showed that if no baseline imbalance is present, all three methods produce similar estimations of an intervention effect, irrespective of pre-post correlations.

When a baseline imbalance is present, however, ANOVA and change analysis both produce biased estimates of the intervention effect, while ANCOVA does not. The direction of the bias (i.e., in favour or against an intervention effect) and the magnitude of the bias varies in complex ways depending on various factors. These factors include whether the baseline imbalance is in the same direction as the intervention or the opposite, the size of this imbalance, whether the pre-post correlation is low/ high and which analysis is being used. Egbewale et al. (2014) also showed that using ANCOVA as the method of baseline adjustment improves both the precision of estimates and also statistical power. For instance, when there is a pre to post-test correlation >=.3, ANCOVA produced more precise parameter estimates than ANOVA and change analysis.

In our data, looking at error scores, we see a significant difference between the pre-test scores of the relevant- and irrelevant-training groups. We also see a pre-post correlation of R = .631, p < .001. Looking at our binding data, no significant baseline difference is seen between groups at pre-test, however, we see a pre-post correlation of R = .520, p < .001. Thus, ANCOVA should be preferred to analyse our results (Egbewale et al., 2014).

**Supplementary Results**

Intentional binding is conceptualised as a *difference* between baseline and operant timing judgments, and is only appropriately considered in conjunction. However, for completeness, we looked at the baseline and operant conditions separately to ensure the post-test differences we saw in binding were not simply the result of changes in the baseline condition only. These tests (see **Table S1A** & **1B**) revealed no post-test differences in baseline measures (p = .909), but a trend towards higher operant values in the relevant training group at post-test (p = .068) – suggesting that, as predicted, changes in the operant condition were largely the basis of the intentional binding group difference.

**Table S1.** Separate comparison of baseline condition and operant condition binding (i.e., not combined into one intentional binding value, as is appropriate).

| Comparison | **A.** ANCOVA: DV post-test baseline; factors Group (relevant- vs. irrelevant training), and pre-test baseline (covariate) | **B.** ANCOVA: DV post-test operant; factors Group (relevant- vs. irrelevant training), and pre-test operant (covariate) |
| --- | --- | --- |
| Group | F(1,35) = 0.13, p = .909, η_p_^2^ = .01 | F(1,35) = 3.55, p = .068, η_p_^2^ = .09 |
| (covariate) Pre-test binding | F(1,35) = 78.94, p < .001,  η_p_^2^ = .69 | F(1,35) = 25.84, p < .001, η_p_^2^ = .43 |

**Supplementary Figures**

**Figure S1.** *Raw error data during testing* ***(left panel)*** *and training* ***(right panel)****. Testing results* ***(left)*** *indicate* *the relevant training group (blue line) improved in performance of the ‘up-left’ thumb movement from pre-to post-test, where the irrelevant training group (red line) did not. This lead significantly lower error for the relevant training group at post-test. While either group trained at performing a different movement (‘up-left’ for the relevant training group, and ‘upward’ for the irrelevant training group) both groups showed a similar amount and rate of learning from the first to last block* ***(right)****. Learning appeared to plateau for both groups after the first block. Please note: drops in motor error indicate improved motor performance. Error bars are standard error of the mean. For raw error data (not pre-post difference) please see* ***Figure 2*** *of the main text.*


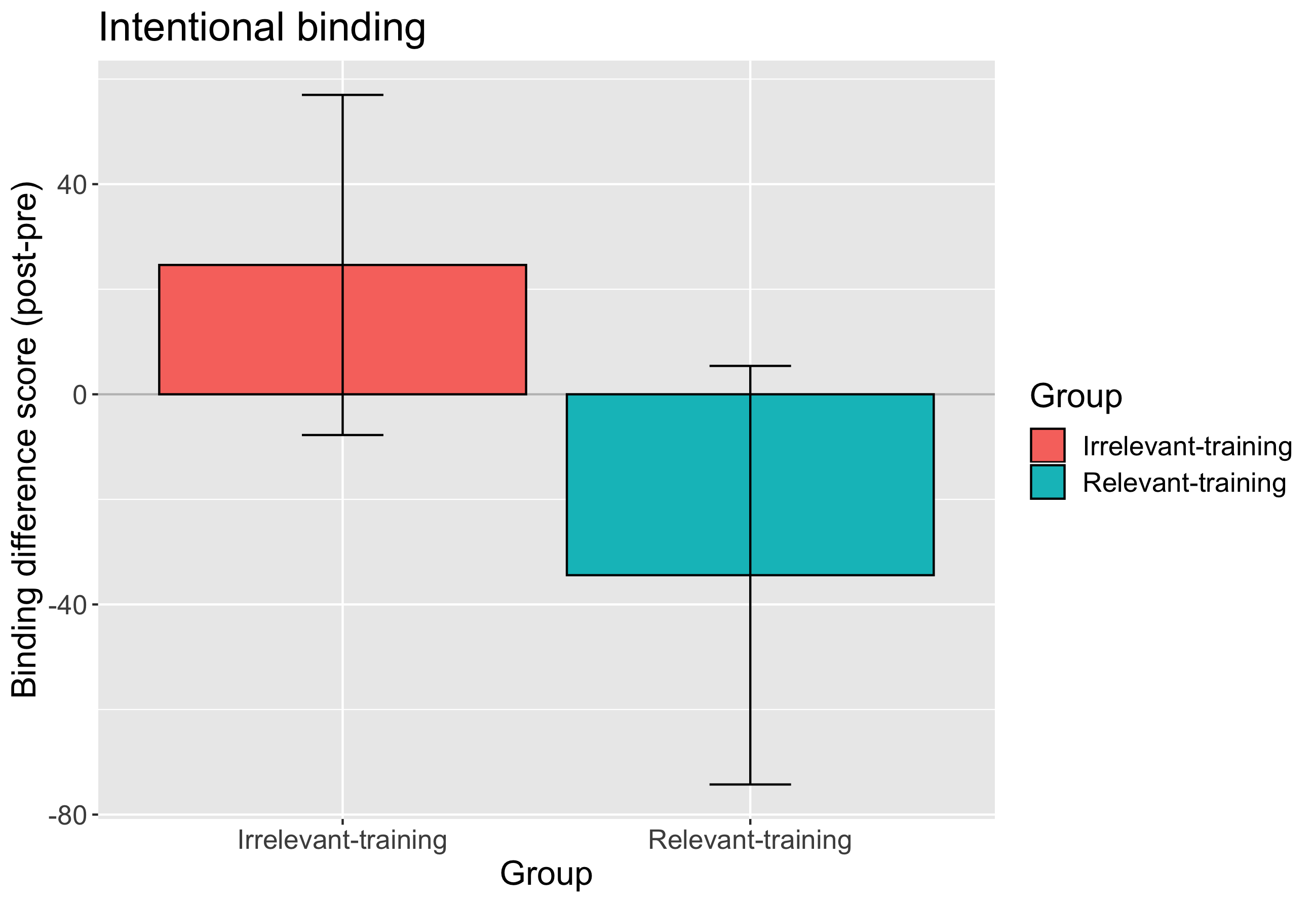


**Figure S2.** *Intentional binding data depicted as change scores (post-pre) for both groups. As can be seen above, the relevant training group showed a relative drop in intentional binding compared irrelevant training group between pre- and post-tests.*

**References**

Galton, F. (1886). Regression towards mediocrity in hereditary stature. *J Anthropol Inst GB Irel., 15*, 246–263.

Huitema. (2011). The Analysis of Covariance and Alternatives: Statistical Methods for Experiments, Quasi-Experiments, and Single-Case Studies. *2nd Edition*.
